# A linear programming model for preserving privacy when disclosing patient spatial information for secondary purposes

**DOI:** 10.1186/1476-072X-13-16

**Published:** 2014-05-29

**Authors:** Ho-Won Jung, Khaled El Emam

**Affiliations:** 1 Korea University Business School, 145, Anam-ro, Seongbuk-gu, Seoul 136-701, Korea; 2 Children’s Hospital of Eastern Ontario Research Institute, 401 Smyth Road, Ottawa, Ontario K1J 8L1, Canada; 3 Pediatrics, Faculty of Medicine, University of Ottawa, Ottawa, Ontario, Canada

**Keywords:** Health services research, Linear programming (LP), De-identified data sets, Geographical identifiers, HIPAA Privacy Rule

## Abstract

**Background:**

A linear programming (LP) model was proposed to create de-identified data sets that maximally include spatial detail (e.g., geocodes such as ZIP or postal codes, census blocks, and locations on maps) while complying with the HIPAA Privacy Rule’s Expert Determination method, i.e., ensuring that the risk of re-identification is very small. The LP model determines the transition probability from an original location of a patient to a new randomized location. However, it has a limitation for the cases of areas with a small population (e.g., median of 10 people in a ZIP code).

**Methods:**

We extend the previous LP model to accommodate the cases of a smaller population in some locations, while creating de-identified patient spatial data sets which ensure the risk of re-identification is very small.

**Results:**

Our LP model was applied to a data set of 11,740 postal codes in the City of Ottawa, Canada. On this data set we demonstrated the limitations of the previous LP model, in that it produces improbable results, and showed how our extensions to deal with small areas allows the de-identification of the whole data set.

**Conclusions:**

The LP model described in this study can be used to de-identify geospatial information for areas with small populations with minimal distortion to postal codes. Our LP model can be extended to include other information, such as age and gender.

## Background

Patients’ geographical identifiers (e.g., geocodes such as postal/ZIP codes, street addresses and locations on maps) are useful for health research and public health purposes [1-4]. Geographical identifiers are also fundamental to the practice of spatial epidemiology [5] and are key components of the public health professional’s toolbox [6].

However, revealing patient data sets, including geographical identifiers, threatens patient privacy if the geographical identifiers can be linked to individuals. In fact, some studies have revealed a threat of re-identification. Sweeney [7] indicated that 87% of subjects could be uniquely identified by their gender, ZIP code and date of birth when linked with other publicly available data, such as voting records. Moskop et al. [8] presented that low-resolution dot maps of diseases published in several medical journals could be used to trace most patients to single addresses. Furthermore, Brownstein et al. [9] also showed that a method of georeferencing and unsupervised classification of the original image could be used to precisely re-identify 26% of 550 patients by using addresses from a presentation quality map and 79% using those from a publication quality map.

The US Health Insurance Portability and Accountability Act of 1996 (HIPAA) allows the disclosure of personal health information for secondary purposes only if the patients provide authorization (with some exceptions) [10]. If it is not practical to obtain authorization then the data must be de-identified before disclosure [11]. Similar laws exist in Canada where de-identification is required for the disclosure of health information without consent [12,13].

According to the HIPAA Privacy Rule, de-identified protected health information (PHI) can be created by one of two ways [10], p.3. The first is the “safe-harbor” method, in which all 18 identifiers, including the five-digit ZIP codes, are removed. Yet, the first three digits of a ZIP code may be included, provided that at least 20,000 people share the same first three digits. The second way is “to have a qualified statistician determine, using generally accepted statistical and scientific principles and methods, that the risk is very small” concerning that such information could be used to identify an individual. The “very small” risk that is used as a threshold for disclosure control depends on the application fields and data users, but has a range of 0.05 to 0.3 of its value [12-15]. This study sets a threshold value of 0.2.

Studies, such as disease mapping or cluster detection in epidemiology, require de-identified data that maximally include the spatial distribution of a disease while complying with a threshold of the re-identification risk. A prevailing method to create de-identified data sets is to aggregate pre-defined areas, such as ZIP codes or counties, into a new area [16]. However, this approach loses useful spatial information while preserving privacy [17]. Furthermore, the level of privacy protection depends on the number of patient records [18]. Another approach uses the deterministic or stochastic function of geographical identifiers [19]. However, this heuristic method cannot quantify the risk to individual privacy and therefore cannot demonstrate that the risk is indeed “very small”.

Wieland et al. [18] proposed a linear programming (LP) model to create de-identified data sets. The LP model determines the transition probability from an original location of a patient to a new randomized location as a de-identification method. However, it cannot be applied to data sets, including locations with small populations (e.g., the population is smaller than the number of patients). For example, the City of Ottawa has 11,740 postal codes that have a population of more than one. Of these, 98.61% (11,577) of postal codes have a population smaller than the number of patients in our data set (224 patients originated from 161 postal codes). The median population in Ottawa postal codes is 10 people.

To apply this LP method on real data sets, where small areas will exist, this study revised the previous LP mode to accommodate the case in which some postal codes can have a smaller population than the total number of patients. The results depicted that our revised model can increase the applicability of the LP model in the creation of de-identified data sets.

## Results

### Results from WCMB-LP model

This study solved two Ottawa LP problems using the WCMB-LP model.

Table 1 shows the range of the maximum re-identification probability and the objective function values. In the table, optimal solutions had the *s*/*ϵ* range of 10 to 33, which corresponds to the *ϵ* value range of 22.4 to 6.79 (where the number of patients, *s*, is 224). That is, WCMB-LP provided impractical optimal solutions for which the re-identification probability is greater than 1.

**Table 1:** WCMB-LP results for the two Ottawa LP problems

**In equation (**3**), ** $\;\nu =\frac{s}{N\cdot \epsilon }=\frac{1}{264,327}\cdot \frac{s}{\epsilon }$		**Objective function value (unit: meter)**	
		**Nearest 10**	**Nearest 30**
$\frac{s}{\epsilon }=10\left(\epsilon =22.4\right)$	*ν* = 3.783 × 10^− 5^	6.0	6.0
$\frac{s}{\epsilon }=20\left(\epsilon =11.3\right)$	*ν* = 7.567 × 10^− 5^	18.9	18.7
$\frac{s}{\epsilon }=30\left(\epsilon =7.47\right)$	*ν* = 11.350 × 10^− 5^	33.3	32.9
$\frac{s}{\epsilon }=33\left(\epsilon =6.79\right)$	*ν* = 12.485 × 10^− 5^	37.5	37.0

### Results from the revised model

We then solved two Ottawa LP problems formulated on the basis of our revised LP model (Revised-LP). Table 2 shows their optimal solutions for the 224 patients. In the nearest 10 dataset, the revised model provided an optimal solution of less than 0.4 of the maximum re-identification probability across all postal code areas. These results were not considered acceptable for preserving privacy across all postal code areas due to the limited transition postal code areas to the nearest 10 neighbors. As described in the Introduction, this study established a threshold value of 0.2 for the re-identification probability.

**Table 2:** Revised-LP results for the two LP problems

**Re-identification probability**	**Objective function value (unit: meter)**	
	**Nearest 10**	**Nearest 30**
*ϵ* = 0.6	321.9	317.9
*ϵ* = 0.5	497.5	478.3
*ϵ* = 0.4	Infeasible*	695.4
*ϵ* =0.3		1,032.1
*ϵ* =0.2		1,686.3

*Note that “Nearest 10” is infeasible when ϵ ≤ 0.4.

When the transition postal code area was extended to the nearest 30, our LP model provided an optimal solution with patient movement of 1,686.3 meter for 0.2 re-identification probability. In this context, we considered that the transition over the nearest 30 would provide a smaller acceptable re-identification probability than the nearest 10. As expected, patient movement was increased for smaller re-identification probabilities, resulting in a greater loss of patient information. As reference, Groubi solution time in a desktop PC (Windows 7 and Intel Core i5 CPUs with 8G RAM) showed less than 4 seconds for Nearest 10 and 8.23 sec to 90 sec (*ϵ* =0.2) for Nearest 30.

## Discussion

Because the area population and latitude and longitude are known for any given postal code, the LP model can generate the optimal transition probability if the number of patients is given. Patient movement in a ZIP code was assumed to follow a multinomial distribution with the transition probability. Thus, two different runs of the same LP problem may provide different patient movements with the same objective function values.

As we have observed in our empirical studies, a limited number of transition neighbors, such as 10, can render LP models infeasible or impractical in terms of achieving an acceptable re-identification probability. However, increasing the transition neighbors greatly increases the computational burden of the LP problem to obtain the optimal solution, considering the postal codes in a country or region. Thus, it is essential to balance a reasonable number of neighbors with consideration for the LP problem size.

## Conclusions

This study expanded the applicability of the previous LP model regardless of the population across all locations (i.e., postal code areas). Thus, our model can be extended to include other information, such as age and gender. Future research may also include a comparison of the performance of our LP model with that of other methods, such as the previously described aggregation methods.

## Methods

### An LP model for de-identified data sets

Wieland et al. [18] introduced an LP model to transform a patient’s spatial identifiers to randomized identifiers in order to create de-identified data sets. In their study, a census block is the only spatial data to be de-identified. Because ZIP codes (postal codes^a^ in Canada) are a common patient residence location indicator [20,21], this study used ZIP codes as a spatial datum to be de-identified. In order to formulate an LP problem, the following notations are defined:

*A*: Set of possible original ZIP codes as identifiers

*B*: Set of possible randomized ZIP codes. This could be different from set *A*

*n*_*i*_: Population in ZIP code *i*

*N*: Sum of populations across all ZIP codes, i.e., ∑ _*i* ∈ *A*_*n*_*i*_ = *N*

*d*_*ij*_: Distance between ZIP codes *i* and *j*

*s*: (Total) number of patients

*ϵ*: Probability that any ZIP code from the randomized dataset originating from any specific individual in the underlying population is at most *ϵ*

*P*_*ij*_: (Decision variable) Transition probability from an original ZIP code *i* ∈ *A* to a new ZIP code *j* ∈ *B*.

Using an LP solution should ensure that the risk is “very small” while minimizing patient movement in order to reduce substantial information loss. With the notations defined, an LP model named WCMB-LP, where WCMB denotes the first character of each of the four authors’ last names in Wieland et al. [18], can be represented as follows:

$$\mathrm{WCMB}‒\mathrm{LP}\left\{\begin{array}{l} \begin{array}{c} \;\mathrm{Min}\sum_{i\in A}\sum_{j\in B}\frac{n_{i}}{N}\cdot d_{\mathrm{ij}}\cdot P_{\mathrm{ij}} \end{array}\\ \;\mathrm{subject}\;\mathrm{to}\\ \begin{array}{c} \;\sum_{j\in B}P_{\mathrm{ij}}=1,\;\mathrm{for}\;\mathrm{all}\;i\in A; \end{array}\\ \begin{array}{cc} \underset{k\in A\;}{\sum }\frac{n_{k}\;}{N}.P_{\mathrm{kj}}-\frac{s}{\mathrm{N\epsilon}}.P_{\mathrm{ij}}\geq 0,\;\mathrm{for}\;\mathrm{all}\;i\in A\;\mathrm{and}\;j\in B; & \;\left(3\right) \end{array}\\ \begin{array}{c} \;P_{\mathrm{ij}}\geq 0,\;\mathrm{for}\;\mathrm{all}\;i\in A\;\mathrm{and}\;j\in B. \end{array} \end{array}\right)$$

The objective function in equation (1) minimizes the expected total movement distance of patients, where *n*_*i*_*/N* denotes a probability that a patient originated from ZIP code *i*. The constraint in equation (2) specifies that the patients in *A* should be moved to somewhere in *B*. This may include self-transition, i.e., patients in a ZIP code may remain there. Constraints in equation (3) implies that “Given the set of *s* locations comprising the de-identified dataset, the probability that any one of these derived from one specific individual to be at most *ϵ*. This is guaranteed if the probability that a location from the randomized dataset originated from an arbitrary specific individual is required to be at most *ϵ*” [18], p. 17612. Further, the transition probability *P*_*ij*_ in equation (4) should be greater than or equal to zero. When the decision variable *P*_*ij*_ is obtained, patients in ZIP code *i* are moved to ZIP code *j* using a multinomial distribution^b^.

Three cases can be investigated to improve the understandability of equation (3) as follows:

[Case 1]: If all ZIP codes include just one person, i.e., *n*_*i*_ = 1 for all *i*, equation (3) becomes *P*_*ij*_ ≤ *ϵ*/*s*.

[Case 2]: If there is just one ZIP code, there is no transition probability, i.e., equation (3) is reduced to *s*/*N* ≤ *ϵ*

[Case 3]: If all patients having a randomized ZIP code *j* are from *i*, i.e., *P*_*kj*_ = 0 for all *k* ≠ *i*, equation (3) becomes *s*/*n*_*i*_ ≤ *ϵ*, where *n*_*i*_ = *N*. This is the same as [Case 2].

### Revised LP model

For a simple interpretation, equation (3) can be rewritten as follows:

(5) $$\begin{array}{cc} \frac{1}{n_{i}}\cdot \frac{\frac{n_{i}}{N}\cdot P_{\mathrm{ij}}}{\sum_{k\in A}\frac{n_{k}}{N}\cdot P_{\mathrm{kj}}}\leq \frac{\epsilon }{s}, & \mathrm{for}\;\mathrm{all}\;i\in A\;\mathrm{and}\;j\in B. \end{array}$$

Equation (5) is the same equation (5) in WCMB-LP [18], p. 17612, where the first part 1/*n*_*i*_ implies the probability that “all individuals in ZIP code *i* with population *n*_*i*_ have an equal chance of having the disease… and the second term is a population-weighted transition probability.”

Equation (5) can also be rewritten as follows:

(6) $$\begin{array}{cc} \frac{s}{n_{i}}\cdot \frac{\frac{n_{i}}{N}\cdot P_{\mathrm{ij}}}{\sum_{k\in A}\frac{n_{k}}{N}\cdot P_{\mathrm{kj}}}\leq \epsilon , & \mathrm{for}\;\mathrm{all}\;i\in A\;\mathrm{and}\;j\in B. \end{array}$$

In equation (6), the right-hand side *ϵ* means that all patients in *B* have the same randomized ZIP code, i.e., a randomized patient list of *s* patients includes one ZIP code. Contrast to equation (5), *s*/*n*_*i*_ denotes a maximum re-identification probability of *s* patients with the same randomized ZIP code, assuming its origination from *i*. In this context, the number of patients cannot exceed the number of people in ZIP code *i*. That is, *s*/*n*_*i*_ ≤ 1.

With these elaborations, equation (3) in WCMB-LP is revised as follows:

(7) $$\begin{array}{ll} \min\left(\frac{s}{n_{i}},1\right).\frac{\frac{n_{i}}{N}\cdot P_{\mathrm{ij}}}{\sum_{k\in A}\frac{n_{k}}{N}\cdot P_{\mathrm{kj}}}\leq \epsilon , & \mathrm{for}\;\mathrm{all}\;i\;\in \;A\;\mathrm{and}\;j\in B. \end{array}$$

Rearranging equation (7) and incorporating it into WCMB-LP, we have the following Revised - LP model (our LP model):

(8) $$\mathrm{Revised}-\mathrm{LP}\left\{\begin{array}{l} \;\mathrm{Min}\sum_{i\in A}\sum_{j\in B}\frac{n_{i}}{N}\cdot d_{\mathrm{ij}}\cdot P_{\mathrm{ij}}\\ \;\mathrm{subject}\;\mathrm{to}\\ \;\sum_{j\in B}P_{\mathrm{ij}}=1,\;\mathrm{for}\;\mathrm{all}\;i\in \;A;\\ \begin{array}{ll} \sum_{k\in A}\frac{n_{k}}{N}\cdot P_{\mathrm{kj}}-\min\left(\frac{s}{\mathrm{N\epsilon}},\frac{n_{i}}{\mathrm{N\epsilon}}\right)\cdot P_{\mathrm{ij}}\geq 0, & \mathrm{for}\;\mathrm{all}\;i\in A\;\mathrm{and}\;j\in B; \end{array}\\ \;P_{\mathrm{ij}}\geq 0,\;\mathrm{for}\;\mathrm{all}\;i\in A\;\mathrm{and}\;j\in B.\; \end{array}\right)$$

Note that equation (8) is the difference between WCMB-LP and our Revised LP.

### Properties of re-identification constraint

In order to investigate the re-identification constraint further, let *v* = min(*s*/*Nϵ*, *n*_*i*_/*Nϵ*) in equation (8). Then, the constraints in equation (8) can be rewritten as:

$$\begin{array}{l} \sum_{k\in A}\frac{n_{k}}{N}\cdot P_{\mathrm{kj}}-\nu \cdot P_{\mathrm{ij}}\geq 0;\\ \begin{array}{cc} \to \sum_{k\in A;k\neq i}\frac{n_{k}}{N}\cdot P_{\mathrm{kj}}+\left(\frac{n_{i}}{N}-\nu \right)\cdot P_{\mathrm{ij}}\geq 0, & \mathrm{for}\;\mathrm{all}\;i\in A\;\mathrm{and}\;j\in B, \end{array} \end{array}$$

where the first part is non-negative because *P*_*kj*_ ≥ 0 for all *k* and *j*, and the second part can be represented by the following function *g* of variable *n*_*i*_:

(9) $$g\left(n_{i}\right)=\left(\frac{n_{i}}{N}-\nu \right)=\left\{\begin{array}{ll} \frac{n_{i}}{N}-\frac{n_{i}}{\mathrm{N\epsilon}}=\frac{n_{i}}{N}\left(1-\frac{1}{\epsilon }\right), & \mathrm{if}\frac{s}{n_{i}}\geq 1\left(i.e.,n_{i}\leq s\right);\\ \frac{n_{i}}{N}-\frac{s}{\mathrm{N\epsilon}}=\frac{1}{N}\left(n_{i}-\frac{s}{\epsilon }\right), & \mathrm{if}\;\frac{s}{n_{i}}<1\left(i.e.,\;n_{i}>s\right). \end{array}\right)$$

**Figure 1:**
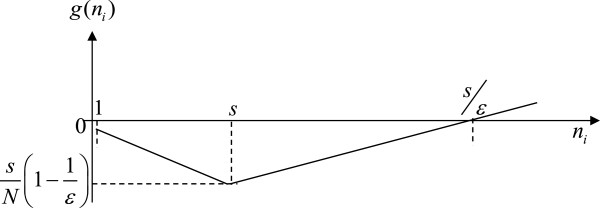
The functional form of equation (9).

Function *g* in equation (9) can be represented as in Figure 1, where *g*(*n*_*i*_) has the smallest value of (*s*/*N*)(1 − 1/*ϵ*) when population *n*_*i*_ = *s* holds and then increases across zero at *n*_*i*_ = *s*/*ϵ*.

The leftmost region from 1 to *s* in Figure 1, i.e., *n*_*i*_ ≤ *s*, corresponds to the first equation in equation (9). In that region, function *g*(*n*_*i*_) always has a negative value and is decreasing because *ϵ* is assumed to be less than 1. The middle region from *s* to *s*/*ϵ*  (i.e., *n*_*i*_ > *s*) corresponds to the second equation in equation (9), where function *g*(*n*_*i*_) is negative and is increasing. The rightmost region (the second equation in equation (9)) denotes that the function *g*(*n*_*i*_) for *n*_*i*_ ≥ *s*/*ϵ* always has a positive value. Thus, the corresponding constraints in equation (8) of our Revised-LP model are always satisfied, i.e., they are redundant because all of its corresponding constraints’ coefficients are nonnegative. A redundant constraint is one that can be left out without changing the model.

### Dataset

In this study, a data set called Ottawa, which includes only areas with a population of more than one, as in Wieland et al. [18], was applied to both WCMB-LP and our Revised-LP models. Our data set was based on patients’ information in a population of 264,327 children under the age of 18 residing in Ottawa, Canada. Our purpose was to randomize the postal codes of a patient list in CHEO (Children’s Hospital of Eastern Ontario) presenting in the emergency department. The patient list included 224 patients from 126 ZIP codes, in which the number of patients corresponded to 5% of an estimated 4,500 people who visited CHEO in a month during the height of the influenza season. The patients were chosen from a pool of CHEO patient postal codes.

The area of each postal code was represented by the centroid latitude and longitude. The distance between two postal-code areas was computed by using the Haversine formula [22], which provides the shortest (also termed ‘as-the-crow-flies’ ignoring any hill or great-circle) distance between any two points on a spherical earth from their longitudes and latitudes. Ellipsoidal effects are ignored, but the result is sufficiently accurate for the purpose of the present study.

Because our data set included 11,740 postal codes, the LP formulation had 137,827,600 variables (i.e., 11,740^2^) and 137,839,340 constraints [i.e., 11,740 (1 + 11,740)]. In order to reduce the size of this LP problem, transitions from any postal code area were limited to the following two cases: the nearest 10 and 30 postal code areas, i.e., two LP problems with 117,400 and 352,200 variables, and 129,140 and 360,940 constraints, respectively. The two LP problems were solved by using Gurobi 6.5.2 solver [23] with MPL 4.2n modeling language [24].

Ethics approval for this study was obtained from the CHEO research Institute research ethics board.

## Endnotes

^a^This study interchangeably uses term “postal” and “ZIP” codes. However, when we mention data from Canada, the term postal codes are intentionally used.

^b^An R library [25] has a command of generating a multinomially distributed random number in *r*. WCMB-LP has |*A*||*B*| variables and |*A*| + |*A*||*B*| constraints.

## Competing interests

The authors declare that they have no competing interests.

## Authors’ contributions

HJ undertook LP modeling, its solution and drafting and revision of the manuscript. KEE participated in the study concept and design, acquisition of data and interpretation of data. All authors read and approved the final manuscript.
